# Case report: Tisagenlecleucel for treatment of relapsed B- acute lymphoblastic leukemia in a patient with *CHEK2* mutation

**DOI:** 10.3389/fped.2023.1067131

**Published:** 2023-03-01

**Authors:** Abraham Ipe, Anne Angiolillo, David Jacobsohn, Jinjun Cheng, Miriam Bornhorst, Joyce Turner, Anant Vatsayan

**Affiliations:** ^1^ School of Medicine and Health Sciences, George Washington University, Washington, DC, United States; ^2^ Department of Leukemia/Lymphoma, Children's National Hospital, Washington, DC, United States; ^3^ Department of Blood and Marrow Transplantation, Children's National Hospital, Washington, DC, United States; ^4^ Department of Hematopathology, Children's National Hospital, Washington, DC, United States; ^5^ Department of Genetics, Children's National Hospital, Washington, DC, United States

**Keywords:** CAR-T, tisagenlecleucel, B-ALL, CHEK2 mutation, MDS

## Abstract

**Background:**

Germline Checkpoint Kinase 2 gene (*CHEK2)* mutations can increase the risk of solid tumors. Recently, they have been identified as risk factors for hematologic malignancies. However, to the best of our knowledge, B-acute lymphoblastic leukemia (B-ALL) has never been described as a presenting manifestation of germline *CHEK2* mutation. Chimeric antigen receptor-T (CAR-T) cell therapy directed against CD19 antigen (tisagenlecleucel) is a novel cellular therapy for treatment of relapsed/refractory (R/R) B-ALL. The use of tisagenlecleucel has not been described in patients with *CHEK2* mutation.

**Case Presentation:**

We describe a case of a pediatric patient with a heterozygous pathogenic germline *CHEK2* mutation (c.1100delC; p.Thr367Metfs*15) successfully treated with tisagenlecleucel for relapsed B-ALL to avoid hematopoietic cell transplant (HCT). The twelve-year-old boy was diagnosed with National Cancer Institute (NCI) high-risk B-ALL (white blood cell count >50,000/mcL), with no extramedullary disease. Cytogenetic analysis revealed normal karyotype but fluorescent *in situ* hybridization (FISH) showed 93% positivity for *CRLF2::P2RY8* rearrangement. He was treated as per Children's Oncology Group (COG) AALL1131 therapy and achieved a complete remission. Seven months after diagnosis, he was found to have papillary thyroid carcinoma with no evidence of metastatic disease. The patient underwent a total thyroidectomy with central lymph node biopsy and radioactive iodine therapy. The patient's biological mother and fraternal twin brother carry the same germline *CHEK2* mutation with no history of malignancy. The biological father tested negative for the familial mutation. The patient's genetic panel also identified three variants of unclear significance: *CDKN2A* (c.37 °C > T; p.Arg124Cys), *FLCN* (c.62G > A; p.Cys21Tyr) and *SDHAF2* (c.139A > G; p.Met47Val). Extended family history also revealed a diagnosis of anaplastic thyroid cancer in maternal uncle at the age of 44 years. Fifteen months after diagnosis the patient had a relapse of B-ALL (both medullary and extramedullary with blasts in CSF), which was successfully treated with tisagenlecleucel. The patient remains in remission 3 years after receiving tisagenlecleucel.

**Conclusion:**

As conventional chemotherapy and radiation can potentially increase the risk of DNA damage and development of secondary malignancies, CD19 CAR-T therapy (tisagenlecleucel) can be used as a substitute for intensive re-induction chemotherapy and HCT in patients with a germline *CHEK2* mutation.

## Introduction

Checkpoint kinase 2 (*CHEK2*) is a tumor suppressor gene that plays a crucial role in the cell cycle and response to DNA damage induced by replication stress and double stranded DNA breaks. CHK2 kinase, a protein coded by the *CHEK2* gene, is also required during mitosis for spindle formation, accurate attachment of kinetochores, and subsequent proper chromosome segregation. Therefore, *CHEK2* mutations can lead to aberrant proteins, contributing to errors in chromosome segregation and a higher frequency of unbalanced structural rearrangements (1). Whether the *CHEK2* gene is a true cancer predisposition syndrome gene by itself remains a topic of debate, but emerging evidence suggests an important role in cancer susceptibility, especially in relation to breast cancers (2, 3). Truncating *CHEK2* gene mutations are well-known pathogenic variants, but the clinical significance of missense variants are subject of ongoing research and their interpretation remains challenging (4). Heterozygous pathogenic germline mutations in *CHEK2* (c.1100delC) have been associated with an increased risk of hereditary breast, prostate, kidney, thyroid, and colon cancers (5–7). Congenital *CHEK2* inactivation is also associated with an increased risk of hematologic malignancies, mainly myeloid neoplasms like myelodysplastic syndrome (MDS) (8). A recent study showed a two times higher frequency of unbalanced structural chromosomal rearrangements (58%) among patients harboring germline *CHEK2* mutations, in comparison to non-carriers (27%) in MDS patients (9). However, lymphoid malignancies, especially B-ALL, seems to be a very rare occurrence in association with *CHEK2* mutations. To the best of our knowledge, there has been no report on the use of tisagenlecleucel for relapsed B-ALL in a patient with a *CHEK2* mutation (c.1100delC). Herein, we report the successful use of tisagenlecleucel for the treatment of early relapsed (both medullary and extramedullary) pediatric B-ALL in a patient with a *CHEK2* mutation and papillary thyroid carcinoma.

## Case presentation

**Figure 1:**
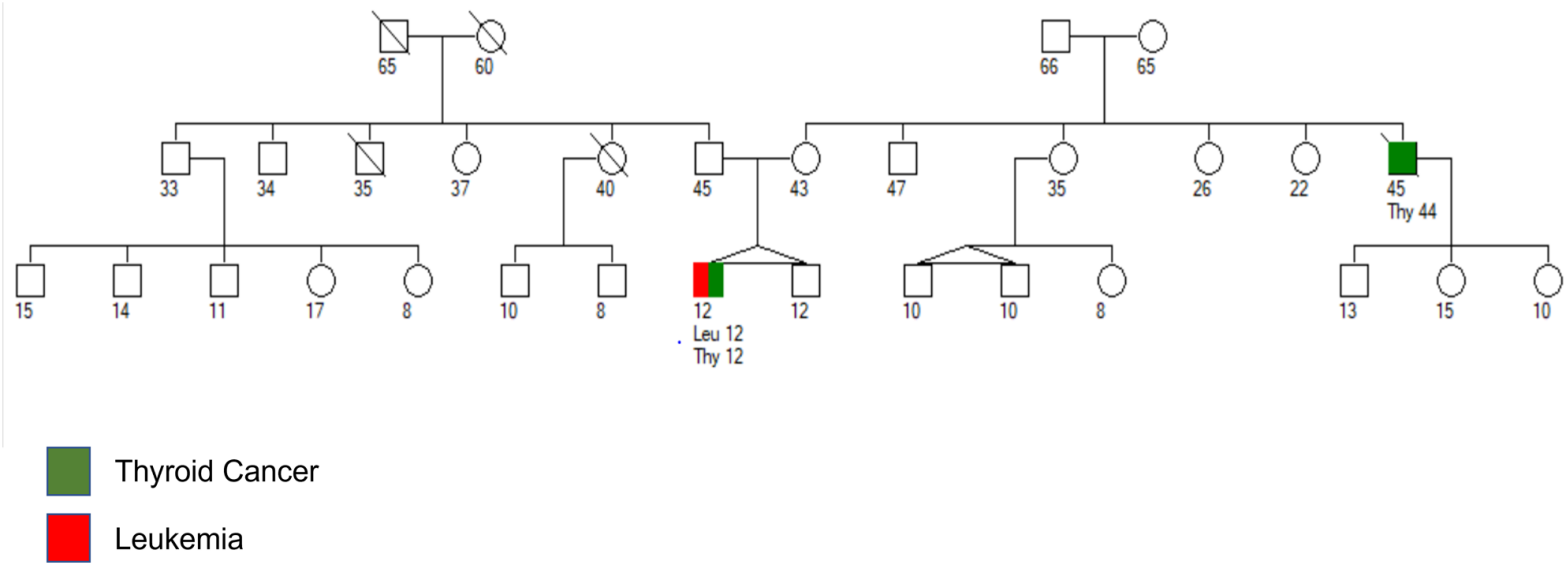
Pedigree chart with age of the patient and family members in years. Maternal uncle was diagnosed with anaplastic thyroid cancer at the age of 44 years and died from metastatic disease at the age of 45 years. Patient was diagnosed with papillary thyroid carcinoma and B-ALL at the age of 12 years.

A 12-year-old boy with a history of hypothyroidism presented with generalized petechiae and hematuria. The patient's white blood count at presentation was 446,750/microliter with mild anemia (hemoglobin 10.6 g/dl), severe thrombocytopenia (24,000/microliter) and 90% B-lymphoblasts in peripheral blood. Lumbar puncture (LP) revealed no blasts in cerebrospinal fluid (CSF). Cytogenetic analysis of bone marrow (BM) blasts revealed a normal karyotype, but fluorescent *in situ* hybridization (FISH) analysis revealed a 93% positivity for *CRLF2::P2RY8* rearrangement. The patient subsequently started therapy as per Children's Oncology Group AALL1131 chemotherapeutic regimen. Family history revealed a diagnosis of metastatic thyroid cancer in a maternal uncle at the age of 44 years who died at the age of 45 years (Figure 1).

**Figure 2:**
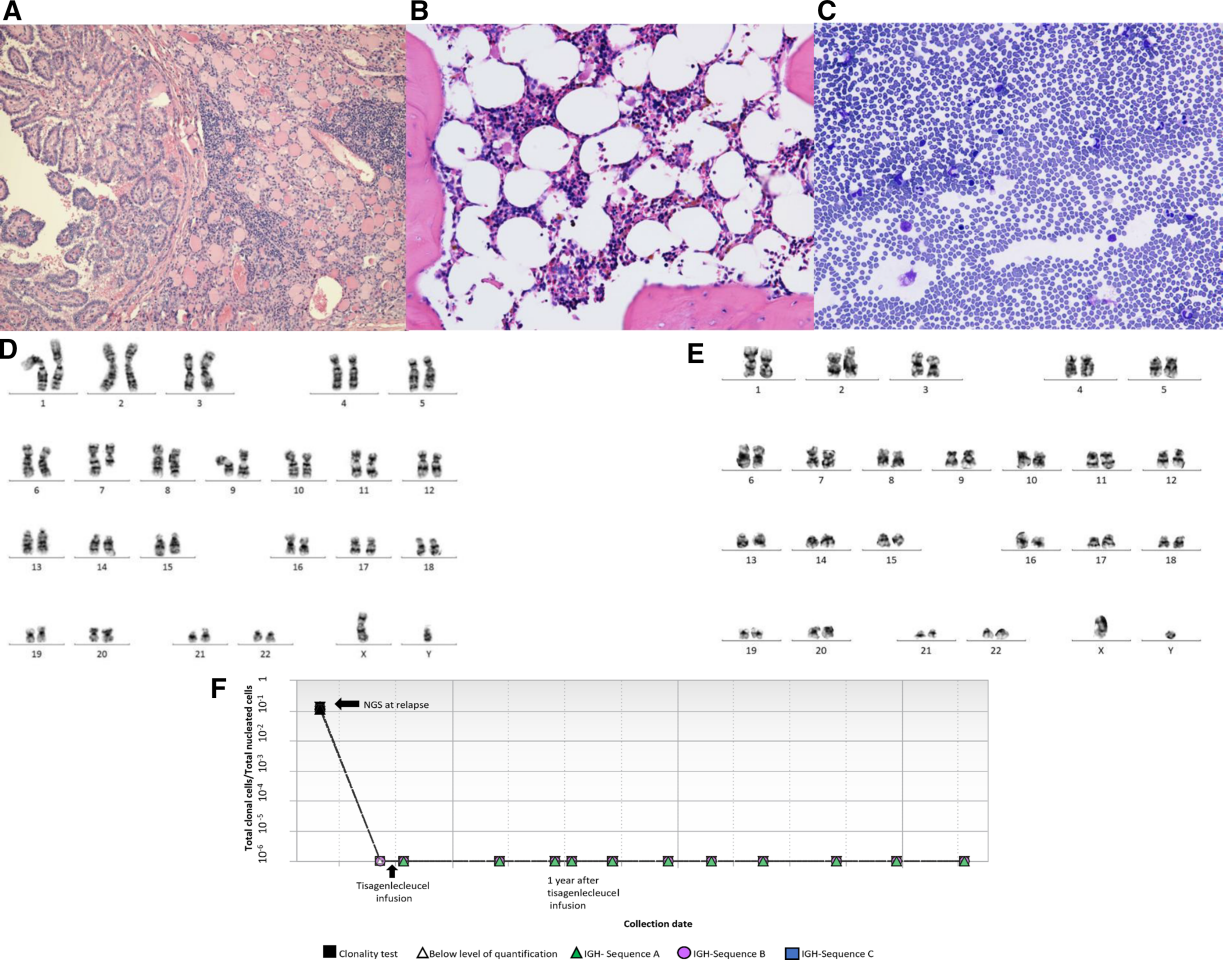
(**A**) Thyroidectomy reveals thyroid tissue with focal chronic inflammation and a papillary carcinoma (H&E stain, 100×). (**B**) Representative bone marrow biopsy reveals hypocellular marrow with trilineage hematopoiesis and no overt dysplasia (H&E stain, 400×). (**C**) Representative bone marrow aspirate reveals few erythroid and granulocytic cells with no overt dysplasia (Giemsa stain, 400×). (**D**) Karyotyping showing deletion of 7q. (**E**) Karyotyping showing absence of deletion 7q in most recent bone marrow specimen. (**F** Next generation sequencing (NGS) showing detection of leukemic clones at relapse and remission (0 residual clonal cells) at 1, 2 and 3 years after receiving tisagenlecleucel.

Seven months after the diagnosis of B-ALL, he presented with a thyroid mass. Fine needle aspiration cytology was diagnostic of papillary thyroid cancer, staged at Bethesda Category V (Figure 2A). Evaluation for metastatic disease was negative. The patient underwent a total thyroidectomy with central lymph node biopsy followed by radioactive iodine therapy. He was referred to Genetics due to concerns for a cancer predisposition syndrome given the development of multiple malignancies within a year. Genetic testing revealed a pathogenic heterozygous *CHEK2* gene mutation (c.1100delC; p.Thr367Metfs*15). The genetic panel also identified three variants of unclear significance: *CDKN2A* (c.37 °C > T;p. Arg124Cys), *FLCN* (c.62G > A; p.Cys21Tyr) and *SDHAF2* (c.139A > G; p.Met47Val). A whole-body MRI was negative for any other malignancies.

Fifteen months after his initial diagnosis, while on maintenance chemotherapy, the patient developed a frontotemporal headache. Computed tomography (CT) scan of the brain showed no intracranial involvement. Mild swelling of optic nerve head was noted on ophthalmologic examination. Increased intracranial pressure and central nervous system (CNS) disease was confirmed by lumbar puncture and magnetic resonance imaging of the brain. Cerebrospinal fluid studies revealed elevated WBC (48/microliter) with 75% lymphoid blasts. Bone marrow aspirate revealed 15% lymphoid blasts by flow cytometry with FISH positive for *CRLF2::P2RY8* rearrangement suggesting relapse with the same leukemic clone that was found on initial diagnosis. Both BM and CSF lymphoid blasts were positive for CD19. He had no signs of any other extramedullary site involvement. He was started on bi-weekly triple intrathecal chemotherapy, in addition to systemic salvage chemotherapy consisting of vincristine, dexamethasone, and mitoxantrone for the next several weeks. The patient was eventually negative for CNS disease sixteen months after initial diagnosis.

**Figure 3:**
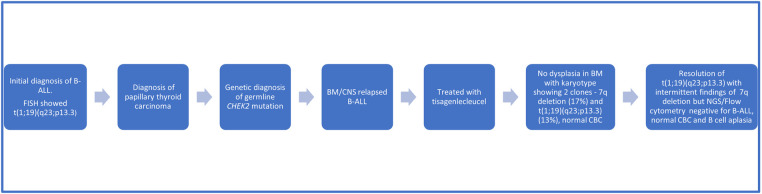
Timeline of clinical events.

In preparation for CAR-T therapy the patient underwent apheresis. The patient was then initiated on a bridging therapy with non-escalating Capizzi protocol prior to receiving tisagenlecleucel. Pre-CAR-T infusion BM and CSF evaluation (done prior to starting lymphodepletion) showed no blasts by flow cytometry. He subsequently received lymphocyte-depleting chemotherapy with fludarabine and cyclophosphamide prior to his infusion of tisagenlecleucel. Four days after CAR-T infusion, the patient developed grade 2 cytokine release syndrome (CRS) but did not develop any immune-effector cell associated neurotoxicity syndrome as per American Society for Transplantation and Cell Therapy Consensus Grading. The patient received tocilizumab and a short course anakinra (3 days). A CSF study was done on day 5 for severe headache in the setting of high-grade fever not responding to tocilizumab, which revealed 70 WBCs/uL with 72% lymphocytes (7% CD19 positive lymphoblasts along with atypical lymphocytes). Day 30 BM evaluation was negative for minimal residual disease by flow cytometry and confirmed by next generation sequencing (NGS) by clonoSEQ (Adaptive). CSF studies at the time showed increased lymphocytes but no blasts. Follow up LPs and BM aspirate/biopsies since then have been consistently negative for lymphoid blasts by flow cytometry. Of note, chromosomal analysis by karyotyping of the patient's bone marrow aspirate, taken 15 months post CAR-T, showed two abnormal clones in metaphase cells. The first clone revealed that 17% of cells had an unbalanced rearrangement of chromosome 7q, which resulted in a partial deletion of 7q, an anomaly frequently associated with primary and secondary myeloid disorders, including MDS. However, no morphologic evidence of myelodysplasia was noted on BM biopsy (Figures 2B,C). The second clone revealed that 13% of cells had a balanced reciprocal translocation (1;19) (q23;p13.3), which results in a TCF3-PBX1 fusion. However, concurrent interphase FISH studies were negative for this fusion. Serial BM biopsies and cytogenetic testing continues to reveal intermittent partial deletion of the distal portion of 7q (Figures 2D,E), while the findings of translocation (1;19)(q23;p13.3) have resolved. His B-ALL remains in remission with no abnormal clones identified on serial NGS testing (Figure 2F) or by immunophenotyping (flow cytometry) and has persistent B cell aplasia. Due to prior history of intensive chemotherapy, germline *CHEK2* mutation and deletion 7q, we suspected a diagnosis of MDS but the follow up CBCs and BM examinations did not confirm our diagnosis due to following reasons. Firstly, the patient's complete blood count improved during the follow up and did not show any progression of cytopenia. Secondly, the chromosomal abnormality of deletion 7q was detected intermittently at a very low percentage that was only detected by karyotyping but not by FISH. Finally, this abnormality was not detected on the patient's most recent bone marrow examination (55 months after initial diagnosis of B-ALL), both on FISH and cytogenetics. We continue to follow this patient closely with serial (every 3- 6 month) CBCs and bone marrow examinations. A summary of the patient's clinical course can be found in Figure 3.

## Discussion

Checkpoint kinase 2 is a tumor suppressor gene that plays a crucial role in the cell cycle and response to DNA damage through the ATM-CHEK2-p53 DNA damage response pathway (1). While truncating loss of function (LoF) mutations are known to be pathogenic, several variants of unknown significance have been described with undetermined functional and clinical significance. Homozygous LoF *CHEK2* mutations can present as Li-Fraumeni (LFS)-like syndrome, whereas heterozygous LoF *CHEK2* variants are moderate penetrance risk factors for solid tumors (2–7). Not surprisingly, emerging evidence also supports the association of certain *CHEK2* gene mutations with myeloid and lymphoid malignancies. LoF *CHEK2* mutations are now increasingly recognized as risk factors for myeloid malignancies (myeloproliferative neoplasms, myelodysplastic syndromes, and acute myeloid leukemia) (8, 9). Interestingly, pre-clinical murine models suggest a role of *CHEK2* mutations (*CHEK2* c.1100delC allele) in development of hematopoietic malignancies besides other solid tumors like breast and lung cancer (10). This is supported by clinical findings in large scale epidemiological studies of these malignant neoplasms (5–7). It must be noted that our patient had other cytogenetic abnormalities and molecular mutations besides *CHEK2* mutation that most likely led to the development of B-ALL, and it is unlikely that the *CHEK2* mutation was the primary driver mutation. However, given the evidence supporting association of *CHEK2* mutation with MDS, the karyotypic findings of clones harboring MDS defining cytogenetic findings (partial deletion of the distal portion of 7q) is concerning for an increased risk of MDS in the future. Therefore, the patient is undergoing close observation with yearly bone marrow examination besides screening for other solid tumors.

The finding of germline *CHEK2* mutation in a patient with relapsed/refractory hematological malignancy has profound clinical implications and presents several challenges. Choosing matched sibling donor (MSD) with the same germline *CHEK2* mutation may present as a clinical dilemma, even though there is no definitive evidence to suggest relapse of leukemia or graft failure after MSD HCT. In our patient we avoided HCT due to availability of CAR-T therapy, the possible need for additional chemotherapy for *CHEK2* mutation-associated solid tumors in the future, and the risk of therapy-related MDS or other secondary malignancies. The use of tisagenlecleucel has been previously described in a patient with LFS and B-ALL, but it has not been described in patients with *CHEK2* mutations (11). Also, the patient did not have a well-matched unrelated donor or umbilical cord blood units. The patient's mother and fraternal twin brother also harbored the same *CHEK2* gene mutation, though the father did not. However, father was not available as a potential donor. Therefore, haploidentical HCT was not considered a viable option. Some reports suggest that relapsed/refractory pediatric B-ALL treated with HCT after CAR-T infusion has significantly better median overall survival than the non-transplant group, especially if post CAR-T NGS is positive even at low levels (12, 13). However, the utility of HCT for patients in deep remission showing no (zero) leukemic clones remains uncertain and could probably be observed without consolidation with HCT (13). Therefore, in our patient with NGS negative remission, we have taken the approach of close monitoring with NGS testing without pursuing HCT. In the future, novel agents like poly (ADP-ribose) polymerase (PARP) inhibitors may be a potential therapeutic option for patients with *ATM* or *CHEK2* mutation associated malignancies (8). The patient's fraternal twin and mother have also undergone genetic counseling and been educated regarding the need for cancer surveillance.

In summary, we describe the case of a pediatric patient with a heterozygous *CHEK2* mutation (c.1100delC (p. Thr367Metfs*15) who presented with high-risk B-ALL and papillary thyroid cancer at the age of 12years. The patient's early relapsed B-ALL with bone marrow and CNS involvement was successfully treated with tisagenlecleucel. The patient responded and tolerated tisagenlecleucel very well. He continues to be in NGS-negative remission 3 years after treatment. The significance of intermittent findings of deletion of 7q on serial BM examinations in the absence of cytopenia and morphologic evidence of dysplasia remains a diagnostic dilemma and does not fulfill the criteria for diagnosis of MDS. However, it is concerning for potential development of either *CHEK2* mutation-associated or therapy-related MDS in future. Hence, we continue to follow this patient closely with serial (every 3- 6 month) CBCs and bone marrow examinations. Moreover, the implication of infused *CHEK2* mutations harbored by autologous CAR-T cells in the long term remains unknown. Our case highlights the need for more research in delineating the true role of pathogenic *CHEK2* gene mutations in hematologic malignancies as a potential cancer predisposition gene. Finally, our case also illustrates that CAR-T therapy can be considered for treatment of R/R B-ALL patients with potential cancer predisposition syndromes such as *CHEK2* mutation, in order to avoid HCT as has been previously described for a patient with LFS who developed B-ALL (11).

## Data Availability

The original contributions presented in the study are included in the article/Supplementary Material, further inquiries can be directed to the corresponding author/s.
